# Editorial: HDL and cardiovascular disease: mechanisms, clinical relevance, and therapeutic impacts

**DOI:** 10.3389/fcvm.2026.1910207

**Published:** 2026-06-26

**Authors:** Jiayou Wang, Yanqiao Zhang

**Affiliations:** Department of Internal Medicine and The Translational Cardiovascular Research Center, University of Arizona College of Medicine - Phoenix, Phoenix, AZ, United States

**Keywords:** biomarkers, cardiovascular disease, HDL, risk prediction, therapeutic implication

High-density lipoprotein-cholesterol (HDL-C) is often referred to as “good cholesterol” because higher levels are associated with a lower risk of cardiovascular disease (CVD). However, attempts to reduce cardiovascular risk by simply increasing plasma HDL-C levels have failed, challenging this traditional view (1–5). Clinical trials using high-dose niacin or cholesteryl ester transfer protein inhibitors, raised HDL-C substantially but failed to reduce CVD (1). In addition, Mendelian randomization studies of genetic variants associated with alterations in HDL levels have not demonstrated a causal association with CVD (3, 6). In contrast, preclinical studies have consistently shown that HDL has direct vascular protective and antidiabetic actions, and diverse mechanisms of favorable action have been demonstrated in multiple cell types, including hepatocytes, macrophages, endothelial cells, and pancreatic *β*-cell (7–10). This has led to the recognition that variations in HDL function likely have great impact on vascular and metabolic health, and more recent research efforts have been placed on enhancing our understanding of HDL mechanisms of action. This Research Topic explores a broader understanding of HDL, highlighting its role in CVD, its biological complexity, and its potential impact on treatment strategies.

Combining HDL with other lipid markers can improve cardiovascular risk prediction. Zhang et al. conducted a cross-sectional clinical study demonstrating that the non-HDL-C/HDL-C ratio is associated with higher abdominal aortic calcification (AAC) scores and a greater risk of severe AAC. In a prospective cohort study, Liu et al. included 43,735 participants and further showed that an elevated cumulative non-HDL-C-to-HDL-C ratio is significantly associated with an increased risk of cardiometabolic disease, independent of single-time-point measurements. Similarly, Luo et al. reported in a prospective cohort study that both the log-transformed platelet-to-HDL-C ratio and its cumulative exposure are independently associated with higher cardiovascular disease risk, and that they provide modest incremental predictive value beyond baseline models. Collectively, these findings support the concept that lipid-related composite indices may outperform HDL-C alone in capturing cardiovascular risk.

Metabolic context beyond traditional phenotypes. Although not directly focused on HDL metabolism, Xie et al. analyzed 4,440 adults with normal body mass index (BMI) and demonstrated significant associations between triglyceride-glucose-related indices and adverse clinical outcomes in individuals with normal BMI. These findings suggest that insulin resistance may be present even in non-obese populations and highlight the need to interpret HDL-related risk within a broader metabolic context.

Genetic and biological complexity of HDL. Sphitzen et al. performed exome sequencing in patients with very low HDL-C, identifying rare genetic variants that may contribute to low HDL-C syndrome. These results underscore the heterogeneity of HDL and emphasize its underlying genetic and biological complexity.

Therapeutic implications. Cao et al. performed a systematic review and meta-analysis demonstrating that the combination of PCSK9 inhibitors and statin therapy significantly reduces major adverse cardiovascular events and improves lipid profiles in patients following percutaneous coronary intervention compared with statin monotherapy. These findings suggest that cardiovascular benefit is achieved through comprehensive lipid modulation rather than targeting HDL alone.

In summary, this Research Topic helps us better understand HDL by showing that it is more than just a simple biomarker and deserves renewed attention. By combining insights from genetics, new biomarker approaches, population studies, and clinical treatments, these studies offer a clearer direction for future research and support better ways to assess cardiovascular risk and guide patient care.
